# Cocaine Self-Administration Elevates GluN2B within dmPFC Mediating Heightened Cue-Elicited Operant Responding

**DOI:** 10.21767/2471-853x.100022

**Published:** 2016-04-22

**Authors:** Karen K Szumlinski, Melissa G Wroten, Bailey W Miller, Arianne D Sacramento, Matan Cohen, Osnat Ben-Shahar, Tod E Kippin

**Affiliations:** Department of Psychological and Brain Sciences & Neuroscience Research Institute, University of California Santa Barbara, Santa Barbara, CA, USA

**Keywords:** Incubation, Craving, Cocaine, NMDA receptor, Prefrontal cortex

## Abstract

Cue-elicited drug-craving correlates with hyperactivity within prefrontal cortex (PFC), which is theorized to result from dysregulated excitatory neurotransmission. The NMDA glutamate receptor is highly implicated in addiction-related neuroplasticity. As NMDA receptor function is regulated critically by its GluN2 subunits, herein, we assayed the relation between incubated cue-elicited cocaine-seeking following extended access to intravenous cocaine (6 h/d; 0.25 mg/infusion for 10 d) and the expression of GluN2A/B receptor subunits within PFC sub regions during early versus late withdrawal (respectively, 3 vs. 30 days). Cocaine-seeking rats exhibited elevated GluN2B expression within the dorsomedial aspect of the PFC (dmPFC); this effect was apparent at both 3 and 30 days withdrawal and occurred in cocaine-experienced rats, regardless of experiencing an extinction test or not. Thus, elevated dmPFC GluN2B expression appears to reflect a pharmacodynamic response to excessive cocaine intake that is independent of the duration of drug withdrawal or re-exposure to drug-taking context. The functional relevance of elevated dmPFC GluN2B expression for drug-seeking was assessed by the local infusion of the prototypical GluN2B-selective antagonist ifenprodil (1.0 µg/side). Ifenprodil did not alter cue-elicited responding in animals with a history of saline self-administration. In contrast, ifenprodil lowered cue-elicited cocaine-seeking, while potentiating cue-elicited sucrose-seeking. Thus, the effects of an intra-dmPFC ifenprodil infusion upon cue reactivity are reinforcer-specific, arguing in favor of targeting GluN2B-containing NMDA receptors as a pharmacological strategy for reducing behavioral reactivity to drug-associated cues with the potential benefit of heightening the reinforcing properties of cues associated with non-drug primary rewards.

## Introduction

The presentation of cocaine-associated cues to human cocaine addicts induces intense drug-craving, increasing the propensity for relapse to drug taking e.g., [1–6]. In cocaine addicts, self-reports of cocaine- craving upon the presentation of drug-related cues or imagery are associated with heightened activation of mPFC [1–3, 6–9]. Thus, it is theorized that a history of excessive cocaine consumption produces neural adaptations within the mPFC that render the addict especially vulnerable to relapse, particularly upon presentation of drug-associated stimuli [10–14]. Indeed, considerable evidence has accrued from studies of animal models of addiction to support the hypothesis that cocaine experience produces enduring anomalies in excitatory signaling within mPFC e.g., [14–20] that may be critical for cue-induced drug-seeking. Studies employing the extinction-reinstatement and the forced abstinence models indicate that cue-elicited cocaine-seeking is paralleled by increased indices of mPFC activation, as indexed by heightened expression of immediate early gene products or by increased phosphorylation of intracellular effectors [21–27]. Direct support for the mPFC in drug-seeking has been derived from neuropharmacological studies demonstrating that transient inactivation of this brain area attenuates cue-induced reinstatement of extinguished behavior [28, 29], and both neuropharmacological and optogenetic approaches have implicated mPFC in the incubation of cue-elicited cocaine-seeking in drug-abstinent animals [24, 30]. In particular, neuronal activity within the more ventral, rather than dorsal, aspects of the mPFC (respectively, vmPFC and dmPFC) appears to be particularly important for the intensification of cue-elicited cocaine-seeking during protracted drug withdrawal [24]. Consistent with this, the vmPFC of cocaine-experienced rats exhibits time-dependent increases in the capacity of drug-paired cues to augment the activational state of several neuroplasticity-related kinases, including extracellular signal-regulated kinase (ERK) [24, 25], and the epsilon PKC isozyme (PKCε) [25], which we hypothesize is driven by an incubation of cue-elicited glutamate release within this region [19].

Relatively recently, a “neural rejuvenation” hypothesis of cocaine addiction was proposed, which states that a history of cocaine exposure induces certain plasticity mechanisms normally associated with brain development within the reward circuitry that mediate the highly efficient and unusually stable memory abnormalities that characterize addiction [31]. *N*-methyl-D-aspartate receptors (NMDARs) receptors play a critical role in the synaptic plasticity underpinning learning and memory processes e.g., [32], including that associated with the acquisition, expression and reconsolidation of cocaine-associated memory e.g., [33, 34]. NMDARs are heterotetrameric proteins, consisting of two compulsory GluN1 subunits and two modulatory GluN2 subunits (A–D) [35]. GluN2 subunits contain the glutamate binding site e.g., [36, 37] and can be differentially regulated by drug experience within several addiction-relevant brain regions, including PFC e.g., [15, 38–40]. Relating back to the “neural rejuvenation” theory, specific GluN2 subunits are developmentally regulated in brain, with GluN2B and GluN2D–containing receptors generally dominating during prenatal and early post-natal development and GluN2A–containing receptors becoming more apparent during entry into adolescent and early adulthood stages [41, 42]. This developmental switch in GluN2 composition markedly affects the functional properties of NMDARs as evidenced by an age-related reduction in: (1) the agonist sensitivity of the receptor for both glutamate and glycine, (2) reduced current flow due to enhanced rates of channel decay imparted by the GluN2A subunit, (3) differential sensitivity to cations and polyamines, as well as (4) differential coupling to intracellular effectors e.g., [36, 37, 41, 43–50]. Thus, adult NMDA receptors are pharmacologically less sensitive to neurotransmitter activation and inactivate more rapidly than those found in juvenile and adolescent animals. As GluN2 subunits are critical for normal cognition c.f., [51–53], this “tuning-down” of NMDA signaling pathways has been implicated in age-related differences in certain types of learning and cognition [54]. Thus, the possibility exists that incubated cocaine-seeking and associated cognitive impairments [18, 25] might reflect a time-dependent “rejuvenation” of NMDA receptors within PFC.

Direct support for this hypothesis is currently lacking in the literature. In fact, only a handful of studies have reported on the effects of repeated cocaine upon the relative expression of GluN2 subunits within the reward memory circuitry [40, 54–59] and the results vary considerably across studies owing to the very divergent cocaine treatment regimens and paradigms employed [40]. For instance, in our hands, a history of extended (6h/day), but not short (1h/day), access to IV cocaine produces time-dependent changes in the expression of GluN2A and GluN2B within mPFC, with an increase in GluN2B and GluN2A expression observed, respectively, at 14 and 60 days withdrawal [16]. Moreover, we showed that a repeated non-contingent cocaine treatment (7×30 mg/kg) up-regulates GluN2A protein expression in the mPFC of both rats and mice when assessed at 3 weeks withdrawal, with mice exhibiting also a trend towards increased GluN2B protein expression [55]. However, a recent report by Liddie and Itzak [40] demonstrated an important interaction between the scheduling/patterning of cocaine exposure and drug context for the detection of a cocaine-induced increase in *Grin2b* and GluN2B, at least within hippocampus. The results of this latter study, coupled with prior evidence that non-pharmacological factors associated with the opportunity to respond for cocaine-associated cues interact with cocaine-taking history to influence the protein expression of glutamate-associated proteins within vmPFC [18, 24, 25, 60], prompted us to examine the relation between cue-elicited cocaine-seeking during withdrawal and the protein expression of GluN2A and GluN2B subunits within mPFC subregions, as well as to assess the functional relevance of increased GluN2B for cue-elicited cocaine-seeking.

## Methods

### Subjects

Male Sprague-Dawley rats (275–325g at the beginning of each experiment) were obtained from Charles River Laboratories (Hollister, CA, USA) and dual-housed in a colony room controlled for temperature (25°C) and humidity (71%), under a 12-hr day/12-hr night cycle (lights on at 20:00 hrs). Animals were given *ad libitum* access to food and water, except during operant training for food reinforcement (see Lever Response Training below). The animals were allowed to acclimate to the colony room for at least 4 days following arrival. All experimental protocols were consistent with the guidelines of the NIH *Guide for Care and Use of Laboratory Animals* (NIH Publication No. 80-23, revised 2011) and were approved by the IACUC if the University of California, Santa Barbara.

### Lever-response training

To engender lever-pressing behavior, rats were first trained to lever-press under a FR1 schedule of food reinforcement (45 mg pellets; Noyes, Lancaster, NH, USA) in sound-attenuated operant conditioning chambers (30×20×24 cm high; Med Associates Inc., St. Albans, VT) during either a 16-hr overnight training session or during daily 1-h training sessions (depending upon availability of our chambers) as described previously by our group e.g., [16–19, 25, 60]. For this, rats were food-deprived for 24 h before the initiation of training and maintained on a restricted diet (5–6 g of food per 100 g body weight per day) for the duration of food training. The operant chambers were equipped with two retractable levers, a stimulus light above each lever, a food pellet dispenser outside the operant box and a food trough between the levers, a house light on the wall opposite to the levers, and a speaker connected to a tone generator (ANL-926, Med Associates). During the session, each lever-press on the active lever resulted in delivery of a food pellet only (i.e., no cues were presented). Lever-presses on the inactive lever had no programmed consequences. Rats that failed to exhibit the criterion of a minimum of 200 responses on the active lever during the overnight session or a minimum of 100 responses on the active lever during 2 consecutive 1-hr sessions received additional lever-response training sessions until criterion was met. Following successful acquisition of lever-pressing behavior (1 or 2 over-night sessions; 5–7 days of 1-h daily sessions), food was available *ad libitum* for the remainder of the study.

### Surgery

Following initial training, animals underwent surgical procedures to implant chronic intravenous (IV) catheters and bilateral guide cannulae into the PFC as described previously by our group e.g., [16–19, 25, 60]. Under ketamine/xylazine anesthesia (56.25 and 7.5 mg/kg, respectively; Abbott Laboratories, North Chicago, IL, USA), rats were implanted with a chronic silastic catheter (13 cm long; 0.3 mm inner diameter, 0.64mm outer diameter; Dow Corning Corporation, Midland, MI, USA) into the right jugular vein. Atropine (0.04 mg/kg) was administered intramuscularly to minimize respiratory congestion during anesthesia and banamine (2 mg/kg; a non-opiate analgesic) was injected subcutaneously to treat post-surgical pain. Each catheter ran subcutaneously around the shoulder to back where it was secured to a threaded 22 gauge metal guide cannula (Plastics One, Roanoke, VA, USA), which emerged from the midline of the animal’s back perpendicular to the dorsal surface. An obturator covered the open end of the cannula to protect from contamination and the cannula was held in place via a small swatch of Bard Mesh (C.R. Bard Inc., Cranston, RI, USA) to which it was cemented. The mesh was, in turn, laid flat subcutaneously on the animal’s back.

Immediately following the IV catheterization and while still under anesthesia, cocaine rats in the behavioral studies were transferred to a stereotaxic apparatus and implanted with 30-gauge guide cannulae (Plastics One Inc.) above the dorsomedial PFC (dmPFC; AP+2.5 mm, ML +/− 1.0 mm, DV −1.0 mm from Bregma), according to the atlas of Paxinos and Watson (2008), using procedures identical to those described previously by our group [18, 25]. Four small screws and cranioplastic cement secured the guide cannulae to the skull. Stylets (Plastics One) were placed into each cannula to prevent occlusion. Sucrose self-administering rats underwent identical intracranial surgical procedures as described for the cocaine animals, but did not undergo IV catheterization surgery. All animals were allowed a minimum of 5 days for recovery and IV catheter patency was maintained by flushing with 0.1 ml of sterile heparin+timentin/saline (60 IU/ml and 100mg/ml, respectively) solution each day.

### Self-administration training

Following surgery, rats (n=10–15 per group at the start of each experiment) were trained to self-administer IV cocaine (0.25 mg/0.1ml/infusion; a generous gift from the National Institute on Drug Abuse, Bethesda, MD, USA) during daily 6-hr sessions on a FR1 schedule of reinforcement. At the start of each session, the rat’s catheter was connected to a mortorized pump (located outside of the sound attenuated chamber) via a liquid swivel as previously described e.g., [16–19, 25, 60]. Active lever-presses resulted in a 5-sec activation of the infusion pump and a 20-sec presentation of a stimulus complex, consisting of activation of the white stimulus light above the active lever and the tone generator (78 dB, 2 kHz), during which responses on the active lever had no consequences. Responses on the inactive lever were recorded, but had no programmed consequences. Rats were trained to self-administer cocaine for 10 sessions and over-dose was prevented by capping the number of cocaine infusions permitted during the first 2 days of training at 100 (day1) and 120 (day2). Rats failing to meet self-administration criterion (minimum of 50 infusions/6-h session for the last 3 days of training) were excluded from the study.

For the immunoblotting study, additional groups of rats were trained to self-administer IV saline (0.1 ml/infusion) during daily 1-h or 6-h sessions (n=12/control group at the start of the experiment) to provide a baseline for determination of cocaine-elicited changes in protein expression [18]. We included both Sal1h and Sal6h animals in our immunoblotting experiments to verify that the duration of the saline self-administration session does not significantly influence NMDA subunit expression of relevance to the design of the neuropharmacological study. As it did not, the neuropharmacological study included Sal1h animals, as well as, an additional group of rats trained to lever-press for 45 mg sucrose pellets (Noyes) during daily 6-h sessions, to examine for the reinforcer-specificity of the effects of intracranial ifenprodil upon cue-elicited behavior. All self-administration training and testing occurred during the dark phase of the circadian cycle. Upon completion of the 10 days of self-administration training, animals remained in their home cages in the colony room until testing for cue-elicited lever-pressing behavior or tissue collection at either 3 or 30 days following the last self-administration session.

### Tests for cue-elicited lever-pressing

In all but one experiment, saline and cocaine self-administering rats were subjected to a test for cue-elicited cocaine-seeking under extinction conditions. For the immunoblotting experiments, the cue tests were 2 h in duration in order to capture potentially relevant changes in receptor protein expression [18]. The details of the descriptive and inferential statistics regarding the behavioral component of the cocaine immunoblotting study are provided in Ben-Shahar et al. [18]. In brief, Coc6h rats emitted a greater number of cue-reinforced active lever-presses than either Sal1h or Sal6h rats during the 2-h test for drug-seeking. Moreover, the analysis of the change in active vs. inactive lever-presses from 3 to 30 days withdrawal indicated an incubation of cue-reinforced responding only in Coc6h rats, as indicated by a time-dependent increase in active lever-pressing without any change in inactive lever-pressing behavior. For the neuropharmacological experiment, the cue tests were 30 min in duration, as conducted previously by our group [18, 25] and by others e.g., [24]. Cue-testing involved tethering the animals fitted with IV catheters, or placing sucrose-trained animals, in the operant chambers, in which the animals received lever-press response-contingent presentation of the tone-light cue previously paired with saline/cocaine infusions or sucrose pellet delivery but no infusion/pellet was delivered.

### Immunoblotting

**Figure 1:**
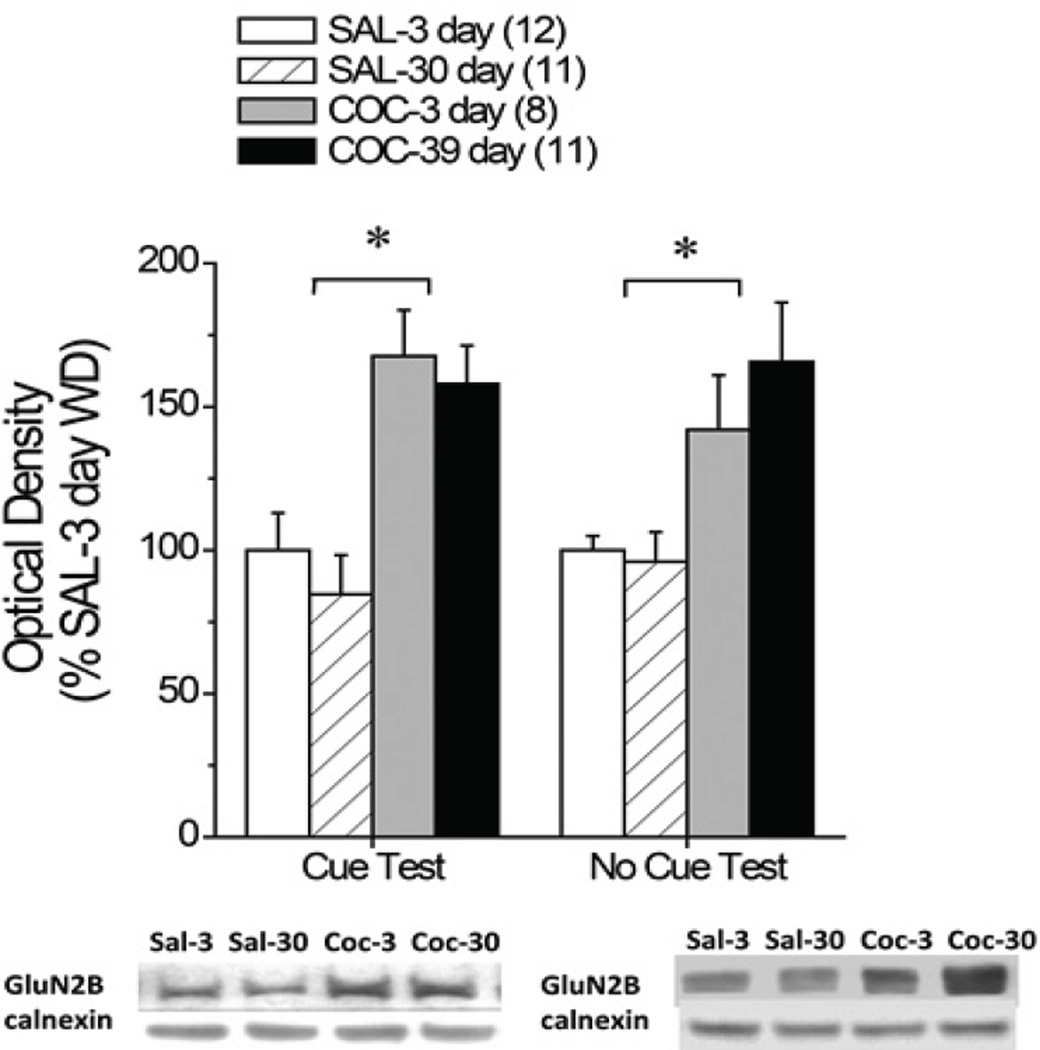
Regulation of GluN2 subunits within PFC subregions during protracted withdrawal from cocaine self-administration and lack of effect of the opportunity to engage in cocaine-seeking behavior Total protein expression GluN2A and GluN2B was determined in the dmPFC of rats with a 10-day history of extended access (6 h/d) to either cocaine (Coc6h) or saline (Sal6h) with or without opportunity to engage in cocaine-seeking behavior under extinction conditions. Relative to Sal6h rats, Coc6h rats exhibited increased levels of GluN2B in the dmPFC regardless of withdrawal extent or opportunity to engage in cocaine-seeking. *p<0.05 vs. Sal6h (LSD post-hoc tests).

The tissue employed for the immunoblotting studies was the same tissue as that immunoblotted for the expression of Group1 mGluRs [18], Homer proteins [60] and PKCε activity [25] and was derived from gross dissections of the vmPFC (infralimbic cortex + ventral prelimbic cortex) and dmPFC (dorsal prelimbic cortex + anterior cingulate cortex) from: (1) groups of Sal1h, Sal6h and Coc6h rats that underwent a 2-h test for cue-reinforced lever-pressing behavior under extinction conductions, conducted at either 3 or 30 days following the end of self-administration training and (2) groups of Sal6h and Coc6h rats that underwent identical self-administration procedures but were not subjected to the behavioral testing procedures (see [18] for details). Rats were sacrificed and tissue collected immediately upon completion of the test session. Protein levels were then assayed by immunoblotting using conventional immunoblotting procedures akin to those described previously by our group e.g., [15, 16, 38, 39, 55, 61–63]. The details regarding the preparation of tissue homogenates, electrophoresis and protein transfer are provided in Ben-Shahar et al. [18]. As conducted previously in our laboratory e.g., [38, 39, 62, 63], rabbit polyclonal antibodies were used to detect GluN2A and GluN2B (Calbiochem, San Diego, CA; 1:1000 dilution). A rabbit primary anti-calnexin antibody (Stressgen, Ann Arbor, MI; 1:1000 dilution) was used as a control to ensure even protein loading and transfer. Membranes were washed, incubated with horseradish peroxidase-conjugated goat anti-rabbit secondary antibody (Millipore; 1:5,000–1:10,000 dilution) for 90 min, washed again, and immunoreactive bands were detected by enhanced chemiluminescence using either ECL Plus (Amersham Biosciences) or Pierce SuperSignal West Femto (Thermo Fisher Scientific, Rockford, IL). Primary antibodies were stripped off PVDF membranes with ReBlot Plus Strong Antibody Stripping Solution (Millipore). Image J (NIH, Bethesda, MD) was used to quantify immunoreactivity levels. An analysis of the immunoreactivity for calnexin indicated even protein loading and transfer e.g., (Figure 1). Thus, the density X area measurements for each protein band was averaged over the appropriate control samples within each gel (n=3–4/gel) and all bands on that gel were expressed as percent of these control values. The immunoblotting data were analyzed using either t-tests or an IV (cocaine vs. saline) X Withdrawal (3 vs. 30 days) ANOVA and significant interactions were deconstructed using t-tests (α=0.05 for all analyses). Pearson correlational analyses were also conducted to determine whether or not a predictive relation existed between cue-reinforced responding and indices of kinase activity within vmPFC and dmPFC of Sal1h, Sal6h and Coc6h rats (α=0.05 for all analyses).

### Microinfusion of ifenprodil

To determine the potential relevance of GluN2B within the dmPFC for drug-seeking behavior, we examined the influence of locally inhibiting GluN2B-containing NMDA receptors using the prototypical antagonist ifenprodil [α-(4-hydroxyphenyl)-β-methyl-4-benzyl-1-piperidine-ethanol(+)-tartrate salt; 1.0 µg/side). This dose of ifenprodil has been demonstrated previously to attenuate alcohol reinforcement when infused into the dorsal striatum [64], as well as to impair fear-related learning when infused into amygdalar or PFC subregions e.g., [65–67] and thus, was deemed behaviorally relevant. Ifenprodil was dissolved in 0.1% DMSO and a 0.1% DMSO infusion served as a control. The procedures for infusing ifenprodil/vehicle were similar to those described for mGlu1/5 antagonists [18]. In brief, drugs were infused via a 33-gauge microinjector cannulae (12 mm long; Plastics One) at a rate of 0.50 µl/min (total volume of 0.50 µl/side) and the injectors remained in place for an additional 60 seconds, at which time, the rats were placed into the operant chambers for a 30-min test for cue-reinforced responding, under extinction conditions. Upon completion of this test, brains were removed and standard histochemical procedures were performed to localize microinjector placements within the dmPFC. The data were analyzed using a Self-administration (saline, sucrose or cocaine) X Treatment (vehicle vs. ifenprodil) ANOVA, followed by post-hoc t-tests when appropriate (α=0.05).

## Results

### Immunoblotting

A history of extended-access IV cocaine self-administration failed to alter the expression of GluN2A within either the vmPFC or the dmPFC, irrespective of the duration of cocaine withdrawal (Table 1). There was no relation between dmPFC or vmPFC GluN2A levels and lever-pressing behavior in saline self-administering rats (n=43; Pearson tests: p’s>0.35). However, a trend for a positive relation between dmPFC GluN2A levels and lever-pressing behavior was noted for cocaine self-administering animals (data not shown; r=0.46, p=0.07; n=17), while there was absolutely no indication of any relation between vmPFC GluN2A expression and cocaine-seeking (data not shown; Pearson test: p=0.96; n=17). A comparable analysis of GluN2B expression within the vmPFC of saline and cocaine self-administering animals also failed to indicate any effect of cocaine upon protein expression (Table 1) and there was no relation between vmPFC GluN2B levels and lever-pressing behavior in either saline (n=43; Pearson test: p=0.82) or cocaine self-administering rats (n=17; Pearson test: p=0.55)

In contrast, dmPFC GluN2B expression was elevated in Coc6h rats at both withdrawal time-points with no evidence for a time-dependent change in protein expression (Figure 1, left) [IV effect: F(1,42)=25.11, p<0.0001; interaction: p=0.85]. Despite the above observations, there were no significant relations observed between dmPFC GluN2B expression and lever-pressing behavior in either saline (data not shown; Pearson test: p=0.45; n=43) or cocaine self-administering animals (data not shown; Pearson test: p=0.76; n=17).

To determine whether or not cocaine self-administration experience alone was sufficient to elevate GluN2B levels within dmPFC, we examined for time-dependent changes in dmPFC GluN2B expression in Coc6h and Sal6h rats that were left undisturbed in the home cage prior to tissue collection. This analysis also revealed a time-independent elevation in dmPFC expression of GluN2b in Coc6h rats (Figure 1, right) [IV effect: F(1,57)=11.82, p=0.001; interaction: p=0.40]. These results indicate that a history of extended access to cocaine is sufficient to elevate dmPFC GluN2B expression and that this effect persists into protracted withdrawal.

### dmPFC infusion of ifenprodil

**Figure 2:**
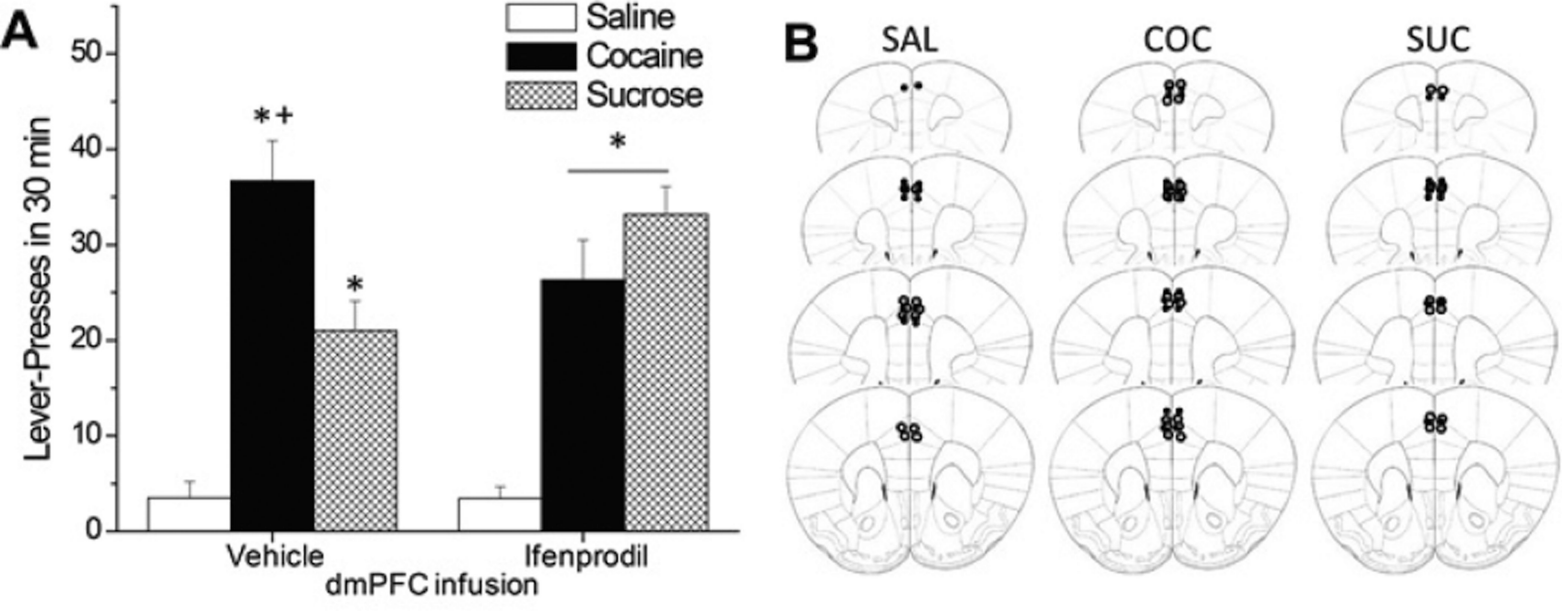
Impact of dmPFC Ifenprodil on cue-elicited responding in saline-, sucrose- and cocaine-trained rats Rats with a 10-day history of extended access (6 h/d) to either saline, sucrose, or cocaine received an intra-dmPFC infusion of the GluN2B antagonist, ifenprodil, or vehicle prior to opportunity to engage in cue-elicited responding at 3 days of withdrawal. **(A).** Cocaine-trained rats exhibited greater lever pressing than sucrose-trained rats following a vehicle, but an ifenprodil, infusion. Both cocaine- and sucrose-trained rats exhibited greater lever pressing than saline-trained rats under both conditions. *p<0.05 vs. Saline; +p<0.05 vs. Sucrose (LSD post-hoc tests).

As correlation cannot inform as to cause-effect relations, we determined the effect of inhibiting GluN2B-containing NMDA receptors within the dmPFC upon cue-elicited lever-pressing in a test conducted at 3 days withdrawal. As expected, the average number of reinforcers earned during the last 3 days of self-administration was greatest in rats trained to lever-press for IV cocaine for 6 h/day or sucrose pellets for 6h/day, relative to IV saline (Table 2) [Reinforcer effect: F(2,43)=13.45, p<0.0001; LSD post-hoc tests: SAL vs. SUC or COC, p<0.0001; SUC vs. COC, p=0.87]. Importantly, there were no group differences with respect to the average number of reinforcers earned between rats slated to receive an intra-dPFC infusion of vehicle vs. ifenprodil for any of the self-administration groups (no main Treatment effect or Treatment X Reinforcer interaction, p’s>0.05) and histochemical evaluation of microinjector placement localized infusion to the intended dorsal aspect of the mPFC (Figure 2B).

On a test of cue-seeking, conducted 3 days following the last self-administration session, the amount of cue-reinforced lever-pressing behavior was found to vary as a function of an interaction between self-administration history and intra-dmPFC treatment [Treatment X Reinforcer : F(2,43)=4.16, p=0.02]. Inspection of Figure 2A suggested an opposite effect of ifenprodil infusion upon cue-elicited responding in cocaine- versus sucrose-trained animals. Thus, follow-up tests for simple main effects confirmed that ifenprodil significantly lowered cue-responsiveness in cocaine-experienced rats (p<0.05); however, the potentiating effect of ifenprodil infusion upon sucrose cue reactivity was not statistically significant (p>0.05). The differential regulation of responding for cocaine vs. sucrose-associated cues, coupled with no apparent effect of ifenprodil infusion upon the behavior of saline-trained animals (p>0.05) argues against the contribution of non-specific motor or cognitive effects to the capacity of intra-dmPFC ifenprodil to reduce cocaine-seeking. Moreover, the results argue a necessary role for GluN2B receptor activity within the dmPFC in regulating behavioral reactivity to cocaine-paired cues.

## Discussion

Using immunoblotting procedures, we show that a history of excessive cocaine intake elevates GluN2B expression within the dmPFC early in drug withdrawal and that this effect persists for at least 30 days following cessation of drug-taking. The cocaine-induced rise in dmPFC GluN2B expression was apparent in cocaine-experienced subjects regardless of whether or not the animals were tested for cue-elicited drug-seeking, indicating that elevated dmPFC GluN2B levels are a pharmacodynamic response to self-administered cocaine and/or cocaine withdrawal. Conversely, no cocaine-induced change in vmPFC GluN2B was observed at either time point and no changes in GluN2A levels were observed within either of the mPFC subregions, arguing against a major role for this subunit within the mPFC in regulating cocaine-seeking or the incubation of cocaine-seeking, at least during the first 30 days of cocaine abstinence.

The functional relevance of elevated dmPFC GluN2B expression for cue-elicited cocaine- as well as sucrose-, seeking during short-term withdrawal was determined by examining the effects of a local infusion of a behaviorally-relevant dose (1.0 µg/side) of the GluN2B-containing NMDA receptor antagonist, ifenprodil [64, 66], and the reinforcer-specificity of ifenprodil’s effect was determined by studying cue-elicited sucrose-seeking, as well as lever-pressing behavior in saline self-administering animals. Consistent with prior studies e.g., [18, 19, 68–70], cocaine- and sucrose-paired cues elicited high rates of lever-pressing behavior, relative to saline-paired cues and this is interpreted to reflect, respectively, cue-elicited cocaine- and sucrose-seeking/craving. Moreover, the efficacy of the cues to elicit operant-responding was increased by pairing with cocaine relative to sucrose, despite equivalent conditioning histories and this is a result in line with findings from other approaches [21, 71]. Thus, the second-order conditioning effects of cocaine are greater than those of natural reinforcers, at least in food/water-sated animals. Although intra-dmPFC ifenprodil did not influence behavior in saline-trained animals, GluN2B blockade attenuated cocaine-seeking and potentiated sucrose-seeking such that cues associated with either reinforcer were equivalent in terms of elicitation of responding (Figure 1A). Despite evidence that GluN2B-containing NMDARs regulate cognitive function e.g., [51–53], the apparent opposing effects of ifenprodil upon cue-elicited cocaine- vs. sucrose-seeking argues against some ubiquitous role for dmPFC GluN2B-containing NMDA receptors in the recall of reinforcer-cue associations or of prior operant learning. Moreover, these data argue that dmPFC GluN2B-containing NMDARs do not play some general role in learning to suppress cue-elicited behavior in the face of null-reinforcer outcomes (i.e., extinction learning), in the motivational properties of secondary reinforcers or in the physical capacity to respond for secondary reinforcers. Whether or not the differential effects of intra-dmPFC ifenprodil upon cocaine- vs. sucrose-seeking reflects reinforcer-specific changes in the complement of GluN2 subunits constituting NMDA receptors within the dmPFC cannot be discerned from the present results as we did not examine for changes in GluN2 subunits in sucrose-trained animals. However, we do know that our training procedures for sucrose and cocaine self-administration result in distinct effects upon cue-elicited glutamate release within mPFC, with sucrose-trained rats exhibiting a cue-elicited rise in extracellular glutamate at 3 days withdrawal, but no change in glutamate at 30 days withdrawal. Conversely, cocaine-trained rats exhibit no cue-elicited change in glutamate during short-term withdrawal, but exhibit a large rise in glutamate when tested at the 30-day withdrawal time-point [19]. Thus, it is clear from our recent work that histories of cocaine- and sucrose-taking produce unique changes in presynaptic aspects of glutamate transmission within PFC. Thus, it seems logical to speculate that the postsynaptic receptors responding to glutamate stimulation would be differentially affected in sucrose-versus cocaine-taking rats. Nevertheless, the present neuropharmacological data support the notion that cocaine-induced increases in GluN2b expression within dmPFC contribute, at least in part, to the manifestation of cue-elicited drug-seeking and do so via mechanism(s) independent of those subserving memory recall, motivation or motor activity.

A direct comparison of subunit expression across the two withdrawal time-points revealed that dmPFC GluN2B expression was elevated in cocaine-taking rats at both 3 and 30 days withdrawal (Figure 1). The present results extend prior work from our group demonstrating an increase in mPFC GluN2B expression in rats withdrawn for 14 days from a history of extended (6h/day) access to IV cocaine [18] and provide greater anatomical insight into the localization of this effect to the more dorsal aspect of the region. GluN2B expression is normally developmentally regulated in brain, with levels lower in adulthood relative to younger individuals [41,42]. Thus, the results of this and our earlier study [16] argue that an extensive history of cocaine-taking induces a switch in the subunit composition of NMDARs to that more typical of earlier development, thereby providing evidence of receptor “rejuvenation” [31] or dematuration. In addition to altering sensitivity to cations and polyamines, the GluN2B subunit confers higher agonist sensitivity and increased channel conductance to NMDARs e.g., [41, 43–50]. Thus, cocaine-experienced animals likely exhibit NMDAR hyperactivity/hypersensitivity within dmPFC. As NMDARs are critical for bursting activity in mPFC neurons in response to reinforcing stimuli e.g., [72], the higher complement of GluN2B-containing NMDARs within dmPFC is predicted to increase the excitability of dm PFC projections to the nucleus accumbens, so critically involved in cue reactivity and cocaine-seeking behavior e.g., [28, 73–75]. This cocaine-elicited change in NMDAR composition (16; present study), coupled with a time-dependent increase in the capacity of cocaine-associated cues to stimulate glutamate release within mPFC [19], could theoretically underpin both cognitive and motivational anomalies in addiction, including pathological psychoreactivity to drug-paired cues.

mPFC GluNB levels normalize by 60 days withdrawal in highly cocaine-experienced rats [16]. Thus, it is not likely that any NMDAR hyperexcitability within dmPFC conferred by elevated GluN2B expression is permanent following a history of excessive cocaine-taking. If anything, the extant data would argue that the opposite might be true as mPFC GluN2A expression is increased markedly, relative to that expressed by saline-taking animals, at 60 days withdrawal in rats with a history of extended cocaine-access [16]. While the present study did not examine for subregional differences in GluNA/B expression at withdrawal times greater than 30 days, these8 NMDAR hypofunction. Consistent with this possibility are data indicating an absence of membrane bistability within mPFC neurons of cocaine-withdrawn rats [20] that is typically generated by the integration of excitatory inputs [76, 77]. While the precise mechanism(s) contributing to the dynamics of GluN2 subunit expression are yet unknown, the recent report by Diddie and Itzak [40] indicated that a history of non-contingent cocaine administration elevates both *Grin2B* mRNA and GluN2B protein expression, at least within hippocampus. Thus, the elevation in GluN2B expression observed selectively within the dmPFC of cocaine-withdrawn rats may very well reflect an increase in both gene transcription and protein translation. Nevertheless, based on what is known regarding the molecular pharmacology of the GluN2B vs. GluN2A subunits, one can speculate how dynamics in the specific GluN2 subunit composition of NMDARs might relate to withdrawal-dependent changes in the excitability of corticocortical and corticofugal projections governing normal executive and cognitive processing to produce the psychobehavioral anomalies characteristic of addiction.

The fact that GluN2 complementation is dynamic during the first few months of cocaine withdrawal [16, 54, 56] (present study) may have important implications for the scheduling NMDAR-targeted pharmacotherapeutics for the treatment of addiction-related cognitive and motivational dysfunction, with inhibition of GluN2B-containing NMDARs being a potential strategy for curbing craving and alleviating other cognitive anomalies during early withdrawal and inhibition of GluN2A–containing receptors being a potential strategy for facilitating cognition and promoting abstinence during more protracted withdrawal. Herein, inhibition of GluN2B-containing NMDARs using a 1.0 µ1/side dose of ifenprodil attenuated, albeit modestly, cocaine cue-elicited drug-seeking when assessed at 3 days withdrawal (Figure 1A). This dose of ifenprodil was selected for the present study as it was demonstrated previously to be effective at reducing alcohol reinforcement when infused into the dorsal striatum [64] and to inhibit the acquisition of a conditioned fear response when infused into the amygdala [66]. However, based on the present immunoblotting results (Figure 1) and results from other studies [16, 40, 54], higher levels of GluN2B-containing NMDAR inhibition are likely required to more effectively overcome the cocaine withdrawal-induced increase in GluN2B expression observed within cortical tissue. Unfortunately, solubility issues precluded our ability to assay the effects of infusing higher drug concentrations and determine whether or not ifenprodil’s effects on both cocaine- and sucrose-seeking are dose-dependent. Given the encouraging outcome of the neuropharmacological study, particularly with respect to the divergent effects of GluN2B blockade upon cocaine- vs. sucrose-seeking (Figure 2A), future work should attempt to extend the current results in both cocaine- and sucrose-experienced animals using higher ifenprodil (and higher DMSO) concentrations or testing varying doses of different, more soluble, GluN2B-containing NMDA receptor antagonists (e.g., Traxoprodil) [78]. Alternatively, given the recent identification of a novel, functionally relevant, binding mode within a subcavity at the GluN1/2 interface for GluN2B antagonists with nonphenylethanolamine scaffolds (e.g., EVT-10) [79], future work could compare and contrast the relative efficacy of GluN2B-directed antagonists with distinct binding modes on GluN2B and/or test whether or not combinations of antagonists with distinct binding modes may be more efficacious at influencing the reinforcing properties of drug and non-drug reinforcers. Finally, based on the fact that systemic treatment with both ifenprodil and Traxoprodil are effective at blocking the acquisition and re-consolidation of cocaine-related memory under place-conditioning procedures [40], the possibility exists that the local blockade of GluN2B-containing NMDARs within the dmPFC alone may be insufficient to completely block the capacity of drug-related cues to elicit craving and drug-seeking behavior and it would be worthwhile in future work to assay the effects of systemic (oral) treatment with GluN2B antagonists, including those with nonphenylethanolamine backbones (e.g., EVT-101) [79], on cue-elicited craving both during early and later withdrawal.

Based on our immunoblotting results, we elected to conduct our neuropharmacological study at an early withdrawal time-point, rationalizing that it was unlikely that the heightened GluN2B levels would differentially contribute to cocaine-seeking during early versus protracted withdrawal (i.e., underpin the development of incubated cocaine-seeking). However, given that a switch in the GluN2 complement of NMDARs appears to occur sometime between 30 and 60 days withdrawal (16; present study), and incubated cocaine-seeking is apparent for at least 60 days withdrawal e.g., [68], it is possible that GluN2B-containing NMDARs within the dmPFC might play a more major role in cue-elicited drug-seeking during protracted versus early withdrawal. Indeed, we have recently reported that an incubation of cue-elicited glutamate release accompanies the incubation of cue-elicited cocaine-seeking [19], but have ruled out the contribution of mGlu1/5 stimulation to the manifestation of incubated responding [18]. Given the important role for NMDARs in neural and behavioral plasticity, future study should seek to determine the relative contribution of GluN2A- vs. GluN2B-containing NMDARs in the incubation of cue-elicited cocaine-seeking during protracted withdrawal, in an effort to understand the neuropharmacology of this relapse-promoting phenomenon and facilitate the design of therapeutics that selectively interfere with the craving-inducing properties of drug-related cues, while sparing (or promoting) the reinforcing properties of non-drug reinforcers.

## Figures and Tables

**Table 1:** Summary of the effects of a history of extended-access to IV cocaine upon the protein expression of GluN2A within both the vmPFC and dmPFC, as well as GluN2b within the vmPFC. As *a priori* comparisons of protein expression failed to indicate differences between Sal1h and Sal6h animals (t-tests, p’s>0.05), the data for the two saline self-administering groups were collapsed (Saline) prior to statistical comparison with that from cocaine self-administering animals. The numbers represent the mean optical density ± SEMs of the number of animals indicated in parentheses.

Protein	Region	3 Days Withdrawal			30 days Withdrawal		
		Saline (n=22–23)	Cocaine (n=6–7)	Statistical Result	Saline (n=22–23)	Cocaine (n=11)	Statistical Result
GluN2A	vmPFC	100 ± 5.38	108.8 ± 3.09	t(26)=0.83, p=0.41	100 ± 3.78	91.60 ± 9.04	t(32)=1.01, p=0.32
	dmPFC	100 ± 3.17	113.38 ± 10.33	t(28)=1.66, p=0.11	100 ± 4.55	113.92 ± 7.50	t(31)=1.67, p=0.11
GluN2B	vmPFC	100 ± 6.01	107.67 ± 14.76	t(26)=0.56, p=0.58	100 ± 2.85	91.93 ± 9.24	t(32)=1.07, p=0.29

**Table 2:** Comparison of the average number of reinforcers earned by rats trained to self-administer IV saline over daily 1-h sessions (Saline), cocaine over daily 6-h sessions (Cocaine) or to self-administer sucrose pellets over daily 6-h sessions (Sucrose) that were slated to receive an intra-dmPFC infusion of DMSO vehicle or 1.0 µg/side ifenprodil. The numbers represent the means ± SEMs of the number of animals indicated in parentheses.

	Vehicle	Ifenprodil
**Saline**	11 ± 3.4 (6)	6.9 ± 1.4 (7)
**Sucrose**	123.1 ± 21.4 (7)+	146.0 ± 29.0 (5)+
**Cocaine**	110.3 ± 9.0 (10)+	161.2 ± 42.5 (10)+

+ p<0.05 vs. Saline (LSD post-hoc tests)

## References

[1] Childress AR, Mozley PD, McElgin W, Fitzgerald J, Reivich M (1999). Limbic activation during cue-induced cocaine craving. Am J Psychiatry.

[2] Childress AR, Ehrman RN, Wang Z, Li Y, Sciortino N (2008). Prelude to passion: limbic activation by "unseen" drug and sexual cues. PLoS One.

[3] Garavan H, Pankiewicz J, Bloom A, Cho JK, Sperry L (2000). Cue-induced cocaine craving: neuroanatomical specificity for drug users and drug stimuli. Am J Psychiatry.

[4] Goldstein RZ, Craig AD, Bechara A, Garavan H, Childress AR (2009). The neurocircuitry of impaired insight in drug addiction. Trends Cogn Sci.

[5] O’Brien CP (2009). Neuroplasticity in addictive disorders. Dialogues Clin Neurosci.

[6] Volkow ND, Wang GJ, Fowler JS, Hitzemann R, Angrist B (1999). Association of methylphenidate-induced craving with changes in right striato-orbitofrontal metabolism in cocaine abusers: implications in addiction. Am J Psychiatry.

[7] Bonson KR, Grant SJ, Contoreggi CS, Links JM, Metcalfe J (2002). Neural systems and cue-induced cocaine craving. Neuropsychopharmacology.

[8] Goldstein RZ, Volkow ND (2002). Drug addiction and its underlying neurobiological basis: neuroimaging evidence for the involvement of the frontal cortex. Am J Psychiatry.

[9] Grant S, London ED, Newlin DB, Villemagne VL, Liu X (1996). Activation of memory circuits during cue-elicited cocaine craving. Proc Natl Acad Sci.

[10] Crunelle CL, Veltman DJ, Booij J, Emmerik-van OK, van den BW (2012). Substrates of neuropsychological functioning in stimulant dependence: a review of functional neuroimaging research. Brain Behav.

[11] Jasinska AJ, Stein EA, Kaiser J, Naumer MJ, Yalachkov Y (2014). Factors modulating neural reactivity to drug cues in addiction: a survey of human neuroimaging studies. Neurosci Biobehav Rev.

[12] Jentsch JD, Taylor JR (1999). Impulsivity resulting from frontostriatal dysfunction in drug abuse: implications for the control of behavior by reward-related stimuli. Psychopharmacology.

[13] Kalivas PW, Volkow N, Seamans J (2005). Unmanageable motivation in addiction: a pathology in prefrontal-accumbens glutamate transmission. Neuron.

[14] Rebec GV, Sun W (2005). Neuronal substrates of relapse to cocaine-seeking behavior: role of prefrontal cortex. J Exp Anal Behav.

[15] Ary AW, Lominac KD, Wroten MG, Williams AR, Campbell RR (2013). Imbalances in prefrontal cortex CC-Homer1 versus–Homer2 expression promote cocaine-seeking behavior. J Neurosci.

[16] Ben-Shahar O, Obara I, Ary AW, Ma N, Mangiardi MA (2009). Extended daily access to cocaine results in distinct alterations in Homer 1b/c and NMDA receptor subunit expression within the medial prefrontal cortex. Synapse.

[17] Ben-Shahar O, Szumlinski KK, Lominac KD, Cohen A, Gordon E (2012). Extended access to cocaine self-administration results in reduced glutamate function within the medial prefrontal cortex. Addiction Biol.

[18] Ben-Shahar O, Miller BW, Sacramento AD, Webb SM, Ditzhazy J (2013). Deficits in ventromedial prefrontal cortex Group1 metabotropic glutamate receptor function mediate resistance to extinction during protracted withdrawal from an extensive history of cocaine self-administration. J Neurosci.

[19] Shin CB, Serchia MM, Shahin JR, Ruppert-Majer MA, Kippin TE (2016). Incubation of cocaine-craving relates to glutamate over-flow within ventromedial prefrontal cortex. Neuropharmacology.

[20] Trantham H, Szumlinski KK, McFarland K, Kalivas PW, Lavin A (2002). Repeated cocaine administration modulates electrophysiological properties of prefrontal cortical neurons. Neuroscience.

[21] Ciccocioppo R, Sanna PP, Weiss F (2001). Cocaine-predictive stimulus induces drug-seeking behavior and neural activation in limbic brain regions after multiple months of abstinence: reversal by D(1) antagonists. Proc Natl Acad Sci U S A.

[22] Hamlin AS, Clemens KJ, McNally GP (2008). Renewal of extinguished cocaine-seeking. Neuroscience.

[23] Hearing MC, Miller SW, See RE, McGinty JF (2008). Relapse to cocaine seeking increases activity-regulated gene expression differentially in the prefrontal cortex of abstinent rats. Psychopharmacology.

[24] Koya E, Uejima JL, Wihbey KA, Bossert JM, Hope BT (2009). Role of ventral medial prefrontal cortex in incubation of cocaine craving. Neuropharmacology.

[25] Miller BW, Wroten MG, Sacramento AD, Silva HE1, Shin CB1 (2016). Cocaine craving during protracted withdrawal requires PKCε priming within vmPFC. Addict Biol.

[26] Neisewander JL, Baker DA, Fuchs RA, Tran-Nguyen LT, Palmer A (2000). Fos protein expression and cocaine-seeking behavior in rats after exposure to a cocaine self-administration environment. J Neurosci.

[27] Zavala AR, Osredkar T, Joyce JN, Neisewander JL (2008). Upregulation of Arc mRNA expression in the prefrontal cortex following cue-induced reinstatement of extinguished cocaine-seeking behavior. Synapse.

[28] Fuchs RA, Evans KA, Ledford CC, Parker MP, Case JM (2005). The role of the dorsomedial prefrontal cortex, basolateral amygdala, and dorsal hippocampus in contextual reinstatement of cocaine seeking in rats. Neuropsychopharmacology.

[29] McLaughlin J, See RE (2003). Selective inactivation of the dorsomedial prefrontal cortex and the basolateral amygdala attenuates conditioned-cued reinstatement of extinguished cocaine-seeking behavior in rats. Psychopharmacology.

[30] Ma YY, Lee BR, Wang X, Guo C, Liu L (2014). Bidirectional modulation of incubation of cocaine craving by silent synapse-based remodeling of prefrontal cortex to accumbens projections. Neuron.

[31] Dong Y, Nestler EJ (2014). The neural rejuvenation hypothesis of cocaine addiction. Trends Pharmacol Sci.

[32] Collingridge G (1987). Synaptic plasticity. The role of NMDA receptors in learning and memory. Nature.

[33] Alaghband Y, Marshall JF (2013). Common influences of non-competitive NMDA receptor antagonists on the consolidation and reconsolidation of cocaine-cue memory. Psychopharmacology (Berl).

[34] Kelley JB, Anderson KL, Itzhak Y (2007). Long-term memory of cocaine-associated context: disruption and reinstatement. Neuroreport.

[35] Zhuo M (2009). Plasticity of NMDA receptor NR2B subunit in memory and chronic pain. Mol Brain.

[36] Kendrick SJ, Lynch DR, Pritchett DB (1996). Characterization of glutamate binding sites in receptors assembled from transfected NMDA receptor subunits. J Neurochem.

[37] Laurie DJ, Seeburg PH (1994). Ligand affinities at recombinant N-methyl-D-aspartate receptors depend on subunit composition. Eur J Pharmacol.

[38] Cozzoli DK, Goulding SP, Zhang PW, Xiao B, Hu J-H (2009). Binge drinking up-regulates accumbens mGluR5-Homer2-PI3K signaling: Functional implications for alcoholism. J Neuroscience.

[39] Cozzoli DK, Courson J, Wroten MG, Greentree D1, Lum EN (2014). Binge alcohol drinking by mice requires intact Group1 metabotropic glutamate receptor signaling within the central nucleus of the amygdala. Neuropsychopharmacology.

[40] Liddie S, Itzhak Y (2016). Variations in the stimulus salience of cocaine reward influences drug-associated contextual memory. Addict Biol.

[41] Monyer H, Burnashev N, Laurie DJ, Sakmann B, Seeburg PH (1994). Developmental and regional expression in the rat brain and functional properties of four NMDA receptors. Neuron.

[42] Yashiro K, Philpot BD (2008). Regulation of NMDA receptor subunit expression and its implications for LTD, LTP, and metaplasticity. Neuropharmacology.

[43] Chen Q, He S, Hu XL, Yu J, Zhou Y (2007). Differential roles of NR2A- and NR2B–containing NMDA receptors in activity-dependent brain-derived neurotrophic factor gene regulation and limbic epileptogenesis. J Neurosci.

[44] Cull-Candy SG, Leszkiewicz DN (2004). Role of distinct NMDA receptor subtypes at central synapses. Sci STKE.

[45] Dingledine R, Borges K, Bowie D, Traynelis SF (1999). The glutamate receptor ion channels. Pharmacol Rev.

[46] Köhr G (2006). NMDA receptor function: subunit composition versus spatial distribution. Cell Tissue Res.

[47] Lynch DR, Guttmann RP (2001). NMDA receptor pharmacology: perspectives from molecular biology. Curr Drug Targets.

[48] Sheng M, Kim MJ (2002). Postsynaptic signaling and plasticity mechanisms. Science.

[49] Sobczyk A, Scheuss V, Svoboda K (2005). NMDA receptor subunit-dependent [Ca2+] signaling in individual hippocampal dendritic spines. J Neurosci.

[50] Stephenson FA (2001). Subunit characterization of NMDA receptors. Curr Drug Targets.

[51] Dumas TC (2005). Developmental regulation of cognitive abilities: modified composition of a molecular switch turns on associative learning. Prog Neurobiol.

[52] Furukawa H, Singh SK, Mancusso R, Gouaux E (2005). Subunit arrangement and function in NMDA receptors. Nature.

[53] Papaleo F, Lipska BK, Weinberger DR (2012). Mouse models of genetic effects on cognition: relevance to schizophrenia. Neuropharmacology.

[54] Tang YP1, Shimizu E, Dube GR, Rampon C, Kerchner GA (1999). Genetic enhancement of learning and memory in mice. Nature.

[55] Ary AW, Szumlinski KK (2007). Regional differences in the effects of withdrawal from repeated cocaine upon Homer and glutamate receptor expression: A two-species comparison. Brain Res.

[56] Hemby SE, Horman B, Tang W (2005). Differential regulation of ionotropic glutamate receptor subunits following cocaine self administration. Brain Res.

[57] Loftis JM, Janowsky A (2000). Regulation of NMDA receptor subunits and nitric oxide synthase expression during cocaine withdrawal. J Neurochem.

[58] Yamaguchi M, Suzuki T, Abe S, Hori T, Kurita H (2002). Repeated cocaine administration differentially affects NMDA receptor subunit (NR1, NR2A-C) mRNAs in rat brain. Synapse.

[59] Zhang X, Lee TH, Davidson C, Lazarus C, Wetsel WC (2007). Reversal of cocaine-induced behavioral sensitization and associated phosphorylation of the NR2B and GluR1 subunits of the NMDA and AMPA receptors. Neuropsychopharmacology.

[60] Gould AT, Sacramento AD, Wroten MG, Miller BW, Klugmann M (2015). Extended access to intravenous cocaine imbalances ventromedial prefrontal cortex Homer1 versus Homer2 expression: Implications for relapse. Addiction Biology.

[61] Goulding SP, Obara I, Lominac KD, Gould AT, Miller BW (2011). Accumbens Homer2-mediated signaling: A factor contributing to mouse strain differences in alcohol drinking?. Genes Brain Behav.

[62] Cozzoli DK, Courson J, Rostock C, Wroten MG, Caruana AL (2016). Extended amygdala protein kinase C epsilon signaling mediates binge alcohol consumption. Biol Psychiat.

[63] Wang J, Carnicella S, Phamluong K, Jeanblanc J, Ronesi JA (2007). Ethanol induces long-term facilitation of NR2B–NMDA receptor activity in the dorsal striatum: implications for alcohol drinking behavior. J Neurosci.

[64] Laurent V, Westbrook RF (2008). Distinct contributions of the basolateral amygdala and the medial prefrontal cortex to learning and relearning extinction of context conditioned fear. Learn Mem.

[65] Rodrigues SM, Schafe GE, LeDoux JE (2001). Intra-amygdala blockade of the NR2B subunit of the NMDA receptor disrupts the acquisition but not the expression of fear conditioning. J Neurosci.

[66] Sotres-Bayon F, Diaz-Mataix L, Bush DE, LeDoux JE (2009). Dissociable roles for the ventromedial prefrontal cortex and amygdala in fear extinction: NR2B contribution. Cereb Cortex.

[67] Grimm JW, Hope BT, Wise RA, Shaham Y (2001). Neuroadaptation. Incubation of cocaine craving after withdrawal. Nature.

[68] Grimm JW, Shaham Y, Hope BT (2002). Effect of cocaine and sucrose withdrawal period on extinction behavior, cue-induced reinstatement, and protein levels of the dopamine transporter and tyrosine hydroxylase in limbic and cortical areas in rats. Behav Pharmacol.

[69] Grimm JW, Fyall AM, Osincup DP (2005). Incubation of sucrose craving: effects of reduced training and sucrose pre-loading. Physiol Behav.

[70] Tunstall BJ, Kearns DN (2016). Cocaine can generate a stronger conditioned reinforcer than food despite being a weaker primary reinforcer. Addict Biol.

[71] Zhang XX, Shi WX (1999). Dendritic glutamate-induced bursting in prefrontal pyramidal cells: role of NMDA and non-NMDA receptors. Zhongguo Yao Li Xue Bao.

[72] Fuchs RA, Eaddy JL, Su ZI, Bell GH (2007). Interactions of the basolateral amygdala with the dorsal hippocampus and dorsomedial prefrontal cortex regulate drug context-induced reinstatement of cocaine-seeking in rats. Eur J Neurosci.

[73] Kalivas PW, McFarland K (2003). Brain circuitry and the reinstatement of cocaine-seeking behavior. Psychopharmacology (Berl).

[74] Peters J, Kalivas PW, Quirk GJ (2009). Extinction circuits for fear and addiction overlap in prefrontal cortex. Learn Mem.

[75] Lewis BL, O’Donnell P (2000). Ventral tegmental area a!erents to the prefrontal cortex maintain membrane potential ‘Up’ states in pyramidal neurons via D1 dopamine receptors. Cereb Cortex.

[76] Steriade M, Nuñez A, Amzica F (1993). A novel slow (< 1 Hz) oscillation of neocortical neurons in vivo: depolarizing and hyperpolarizing components. J Neurosci.

[77] Chenard BL, Bordner J, Butler TW, Chambers LK, Collins MA (1995). (1S,2S)-1-(4-hydroxyphenyl)-2-(4-hydroxy-4-phenylpiperidino)-1-propanol: a potent new neuroprotectant which blocks N-methyl-D-aspartate responses. J Med Chem.

[78] Kerstetter KA, Aguilar VR, Parrish AB, Kippin TE (2008). Protracted time-dependent increases in cocaine-seeking behavior during cocaine withdrawal in female relative to male rats. Psychopharmacology.

[79] Loweth JA, Scheyer AF, Milovanovic M, LaCrosse AL, Flores-Barrera E (2014). Synaptic depression via mGluR1 positive allosteric modulation suppresses cue-induced cocaine craving. Nat Neurosci.

